# Analyzing the intersection of race and income on perceived discrimination among sexual and gender diverse persons in Brazil: a nationwide cross-sectional study

**DOI:** 10.1186/s12889-026-27313-4

**Published:** 2026-04-15

**Authors:** Thiago S. Torres, Lucilene Freitas, Mayara S. T. Silva, Caroline Guimarães, Karine M. Barreto, Brenda Hoagland, Valdilea G. Veloso, Beatriz Grinsztejn, João L. Bastos, Paula M. Luz

**Affiliations:** 1 Instituto Nacional de Infectologia Evandro Chagas, Fundação Oswaldo Cruz (INI-Fiocruz), Av. Brasil 4365, Manguinhos, Rio de Janeiro, 21040-900 Brazil; 2 Escola Nacional de Saúde Pública Sérgio Arouca, Fundação Oswaldo Cruz (ENSP-Fiocruz), Rio de Janeiro, Brazil; 3 Faculty of Health Sciences, Simon Fraser University (FHS/SFU), Burnaby, BC Canada

**Keywords:** Intersectionality, Discrimination, Race, Income, Sexual and Gender Minorities; Men Who Have Sex with Men (MSM), Transgender and Non-Binary persons, Brazil, Latin America

## Abstract

**Background:**

Existing research shows that sexual and gender diverse populations living in the Global South are systematically discriminated against based on sexual orientation and/or gender identity. Knowledge remains limited, however, on how race and class individually and intersectionally shape perceived discrimination. We analyzed whether and how race and income intersect to shape perceived discrimination among sexual and gender diverse persons.

**Methods:**

Our data come from an online survey with sexual and gender diverse persons ≥ 18 years living in Brazil, between November/2021 and January/2022. We used the 8-item Explicit Discrimination Scale to assess the frequency of perceived discrimination (higher scores=higher discrimination), including the self-reported attributions. Participants were stratified into six mutually exclusive groups considering race (Black/Pardo/White) and income (Low/High). Negative binomial regression models were used to assess variables associated with discrimination.

**Results:**

In total, 7878 participants were included, 49.4% high-income White, 18.4% high-income Pardo, 11.1% low-income White, 8.3% low-income Pardo, 8.1% high-income Black and 4.7% low-income Black. High-income White had the lowest discrimination score whereas Black participants had the highest scores regardless of income, with low-income Black participants reporting the highest number of attributions. Sexuality-based discrimination was perceived by all groups to a similar degree, regardless of race and income. Model results revealed significant intersectional patterns of race and income in discrimination experiences, even after adjusting for interaction terms of race/income and other co-variables. In the adjusted model, the joint effect analysis showed that, compared to high-income White participants, discrimination scores were 1.22 (95% CI: 1.09-1.36), 1.37 (95% CI: 1.27-1.49), 1.98 (95% CI: 1.69-2.32), and 1.78 (95% CI: 1.63-1.96) times higher among high-income Pardo, low-income Pardo, high-income Black and low-income Black participants.

**Conclusions:**

Our findings underscore the importance of assessing perceived discrimination according to an intersectionality perspective. Results show the pervasive and multifaceted nature of discrimination experienced by sexual and gender diverse persons in Brazil, with disparities according to race and income. The findings highlight the compounded disadvantage faced by Black and low-income individuals, who perceive heightened levels of discrimination across multiple domains. Targeted efforts are needed to mitigate discrimination faced by racially and economically marginalized sexual and gender diverse populations.

## Background

In Brazil, much progress has been made to promote human rights and counteract discrimination based on sexual orientation and gender identity among lesbian, gay, bisexual, transgender, queer, intersex, asexual, pansexual, non-binary, plus (LGBTQIAPN+) or sexual and gender diverse populations. Same-sex couple adoption was approved in 2010, same-sex marriage in 2013, and a law that criminalizes discrimination based on sexual orientation and gender identity was passed in 2019 [1]. Despite these advances, human rights in the country are neither guaranteed nor stable. In 2023, a conservative group from the Brazilian Congress approved a bid to prohibit the legal recognition of same-sex marriage. Additionally, Brazil is a violent country for sexual and gender diverse populations, with the highest number of registered homicides based on sexual orientation or gender identity worldwide [2]. In 2025, under the pretext of updating medical guidelines, the Federal Council of Medicine banned hormone prescriptions for trans people, a scientifically unfounded and ethically indefensible decision that reflects the growing influence of conservative forces and deepens structural violence against sexual and gender diverse communities [3].

Beyond sexuality- and gender-based mistreatment, sexual and gender diverse populations can also be subjected to other forms of discrimination based on, for instance, race and social class, especially where other systems of oppression co-exist and intersect. In 2023, the richest 10% of the Brazilian population had an income 14.4 times higher than that of the 40% with the lowest income [4]. The Brazilian society has historically been shaped by colonialism and slavery, where race- and class-based discrimination continue to target specific population groups, providing them with significantly fewer opportunities, privileges and rights, including the right to health [5]. Racism predisposes Black Brazilians to social vulnerability, limiting educational and employment opportunities, while increasing psychosocial stress and the risk of experiencing environmental injustices [6]. Ultimately, racism undermines access to healthcare, leading to poorer health outcomes for Black Brazilians compared to their White counterparts [7].

As articulated by Kimberlé Crenshaw, intersectionality provides a critical framework for understanding how multiple systems of oppression intersect to produce unique and compounded forms of marginalization for specific groups and individuals [8]. Recent studies in Brazil have embraced this perspective by using instruments that measure perceived discrimination across intersecting axes such as race, social class, gender, and sexual orientation [9–11]. This marks a significant shift from earlier research on sexual and gender diverse populations, which tended to focus narrowly on discrimination related to sexual orientation or gender identity [12–14], without considering race, social class, or the intersections among these and other systems of oppression.

These intersecting forms of discrimination appear linked to broader processes of structural vulnerability, which are influenced by prevailing socioeconomic systems. Historical and contemporary social structures, shaped by factors such as colonialism, racism, classism, and heterosexism, may contribute to the systematic production and reproduction of inequalities [15, 16]. Such systems can result in the marginalization of populations situated at the confluence of multiple axes of discrimination, such as sexual and gender diverse persons of Black race from lower socioeconomic strata. Conceptualizing vulnerability not as an intrinsic characteristic but rather as a consequence of these structural dynamics is pertinent for analyzing barriers to health and well-being [17, 18].

Our group conducted a study to examine whether and how different forms of discrimination are reported by sexual and gender diverse persons living in Brazil. We not only found that sexual and gender diverse persons self-identified as Black reported the highest frequencies of perceived mistreatment but also that race-based mistreatment, rather than LGBTQIAPN+phobia, was perceived as the primary attribution for the experiences of mistreatment [19]. In the present analysis, we analyzed whether and how race and income intersect to shape perceived discrimination among sexual and gender diverse persons living in Brazil.

## Methods

### Study design

**Fig. 1:**
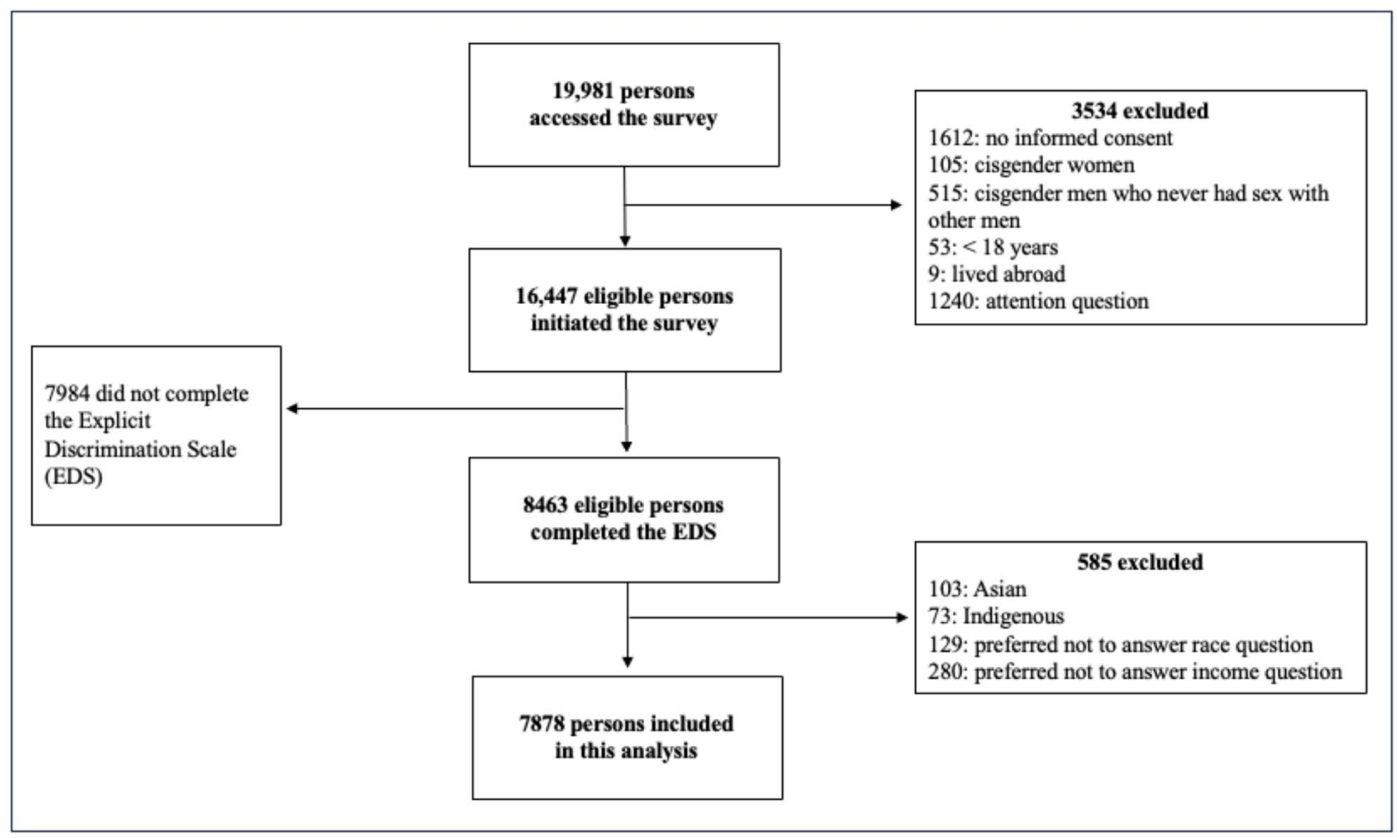
Study flow-chart

This is a secondary analysis of data coming from a cross-sectional, internet-based survey among sexual and gender diverse persons living in Brazil. Details of the study design were reported elsewhere [19]. Briefly, recruitment was conducted on dating apps (Hornet, Scruff and Grindr) and social media (Facebook and Instagram) from November 2021 to January 2022. Requests for participation in the study were sent through direct messaging or boosted posts, depending on the platform. After providing electronic informed consent, participants were directed to the survey, which was administered via Alchemer^®^ (https://www.alchemer.com/). Only one response per internet protocol (IP) address was allowed and no compensation for participation was provided, as per Brazilian regulations. Participants were excluded if they: (a) self-identified as cisgender woman or cisgender man who never had sex with men; (b) reported living outside of Brazil; (c) incorrectly answered any of the five attention checks; [20] or (d) did not complete the questionnaire (Fig. 1). Our analytical sample excluded respondents with no information on race (*n* = 129) and income (*n* = 280), as well as those who identified as Indigenous (*n* = 73) or Asian (*n* = 103).

### Dependent variable

Lifetime perceived discrimination was assessed with the Explicit Discrimination Scale (EDS) [11]. Developed for use in Brazilian large-scale surveys, the EDS was originally composed of 18 items on perceived discrimination in different day-to-day settings, along with questions on the participants’ perceived attributions for each of these experiences. For the present analysis, we used a shortened version of the EDS with 8 items, the psychometric properties of which have been shown to be acceptable among working-age community respondents [9]. For each of the 8 items, participants responded to four questions. First, they were asked the frequency with which they were mistreated in a specific interpersonal interaction. The response options were (response [coding]): never (0), occasionally (1), frequently (2), and always (3). Participants first identified the primary reason why they believed they experienced mistreatment from a list of 15 categories (e.g., race, social class, sexual orientation, gender, disease, age, housing location, accent, clothing, overweight, physical disability, appearance, political beliefs, religion, or other). They then had the option to select additional attributions from the same list. Finally, respondents indicated whether they considered the mistreatment to be discriminatory (Yes/No). For those who answered “Yes,” the responses from the initial eight items were summed to generate a discrimination score ranging from 0 to 24, with higher scores reflecting more frequent experiences with discrimination.

### Main explanatory variables

Race was classified according to the recommendations by the Brazilian Institute of Geography and Statistics (Instituto Brasileiro de Geografia e Estatística, IBGE) into: Black, which includes people who identify as Black or of African descent; Pardo, which includes people who identify as mixed race or multiracial, with a combination of Black, Indigenous or White ancestry; and White, which includes people who identify as White or of European descent. Income was based on household monthly income, assessed in relation to the minimum monthly wage, which was BRL 1,212 in 2021, equivalent to USD 212. We dichotomized income into “low” (2× the minimum wage or lower) and “high” (more than 2× the minimum wage), based on the threshold for tax exemption in Brazil [21]. A variable combining race and income was created, resulting in the following 6 mutually exclusive groups: high-income White, low-income White, high-income Pardo, low-income Pardo, high-income Black and low-income Black.

### Co-variables

Participants also provided information on age, gender, sexual orientation, education, region of residence, whether they live in a state capital, and HIV status. Response options for gender included cisgender man, cisgender woman, transgender man, transgender woman, *travesti* and non-binary or gender diverse/non-confirming person. Response options for sexual orientation included gay or homosexual, bisexual, heterosexual and other (e.g., pansexual, asexual, or demisexual). Education was categorized as elementary or lower (9 years or less), secondary (10–12 years), and higher than secondary (more than 12 years). Respondents’ state of residence was grouped into the country’s five regions, based on the official administrative definition: north (seven states), northeast (nine states), center-west (three states and federal district), southeast (four states), and south (three states). HIV status was self-reported and classified as negative, positive or unknown (includes those who never tested or who did not know their test result).

### Statistical analysis

First, a description of the sample in absolute and relative frequencies (categorical variables) or by median and interquartile range (continuous variables) was provided overall and according to the 6 race-by-income groups. Next, we estimated the percentage of participants who reported discrimination, dichotomized into “No” (never) and “Yes” (occasionally, frequently or always). Then, we described the attributions for discrimination, and the mean number of attributions along with the standard deviation. Statistical significance was assessed using chi-square test or ANOVA f-test. Finally, the mean EDS score was calculated overall and by race/income group. We calculated the ratio of means for each group in relation to the reference group (high-income White participants) and compared the mean discrimination score of each group with the reference group score using the t-student test.

We then used negative binomial regression models to evaluate the association between the main explanatory variable (race/income groups) and the dependent variable (discrimination score). The binominal regression model was preferred over other options as preliminary analyses indicated that the distribution of the discrimination score was over-dispersed (variance > mean) [19]. In Model 1, we included only the main explanatory variables and an interaction term between race and income. Model 2 further added the co-variables age, gender, sexual orientation, education, Brazilian region, living in state capital and HIV status. Instead of providing separate main effects for race and income plus the effects for the interaction terms, we provide the joint effects by quantifying race-income group contrasts, assuming high-income White participants as the reference category. Model coefficients were exponentiated to yield incidence rate ratios (IRRs) that represent the multiplicative effects on the mean discrimination score, e.g. IRRs compare mean discrimination scores between categories of the variables included in the model. We also present a graphical representation of the joint effect of race and income on the discrimination score created using the “effects” package in R. A *p*-value of 0.05 or less indicated statistically significant associations. All statistical analyses were performed using R software, version 4.4.1.

## Results

A total of 7,878 participants were included in this study, predominantly recruited through Hornet (44.3%), followed by Scruff (26.0%) then Grindr (21.6%) (Table 1). The median age was 36 years (IQR: 29–44), with most respondents self-identifying as cisgender man (97.8%), and reporting sexual orientation as gay/homosexual (83.8%). A large proportion of participants reported higher than secondary education (74.0%), living in the southeast (69.8%), and in a state capital (72.6%). Self-reported HIV status was as follows: 67.0% negative, 26.1% positive and 6.9% unknown.

**Table 1:** Socio-demographic characteristics overall and stratified by race/income groups in the cross-sectional, internet-based survey among sexual and gender diverse persons in Brazil, November 2021 to January 2022

	Total	White		Pardo		Black	
		High-income *n* (%)	Low-income *n* (%)	High-income *n* (%)	Low-income*n* (%)	High-income *n* (%)	Low-income *n* (%)
	7878	3894 (49.4)	874 (11.1)	1451 (18.4)	654 (8.3)	635 (8.1)	370 (4.7)
Age (years)							
Median (IQR)	36 (29,44)	37 (31,46)	33 (27,42)	36 (30,43)	32 (27,40)	34 (29,41)	29 (25,36)
18-24	741 (9.4)	276 (7.1)	123 (14.1)	101 (7)	111 (17)	53 (8.3)	77 (20.8)
25-35	3166 (40.2)	1394 (35.8)	388 (44.4)	585 (40.3)	303 (46.3)	305 (48)	191 (51.6)
>35	3971 (50.4)	2224 (57.1)	363 (41.5)	765 (52.7)	240 (36.7)	277 (43.6)	102 (27.6)
Gender							
Cisgender man	7704 (97.8)	3833 (98.4)	836 (95.7)	1432 (98.7)	631 (96.5)	618 (97.3)	354 (95.7)
Transgender man	19 (0.2)	4 (0.1)	5 (0.6)	2 (0.1)	5 (0.8)	2 (0.3)	1 (0.3)
Transgender woman	15 (0.2)	3 (0.1)	4 (0.5)	1 (0.1)	3 (0.5)	2 (0.3)	2 (0.5)
* Travesti*	8 (0.1)	2 (0.1)	1 (0.1)	1 (0.1)	4 (0.6)	0 (0)	0 (0)
Non-binary person	132 (1.7)	52 (1.3)	28 (3.2)	15 (1)	11 (1.7)	13 (2)	13 (3.5)
Sexual Orientation ^1^							
Gay or Homosexual	6598 (83.8)	3366 (86.5)	740 (84.8)	1199 (82.8)	500 (76.5)	511 (80.6)	282 (76.4)
Bisexual	1034 (13.1)	423 (10.9)	106 (12.1)	214 (14.8)	126 (19.3)	100 (15.8)	65 (17.6)
Heterosexual	73 (0.9)	33 (0.8)	9 (1)	11 (0.8)	9 (1.4)	5 (0.8)	6 (1.6)
Other ^2^	165 (2.1)	70 (1.8)	18 (2.1)	24 (1.7)	19 (2.9)	18 (2.8)	16 (4.3)
Education ^3^							
Elementary or lower	205 (2.6)	42 (1.1)	48 (5.5)	23 (1.6)	58 (8.9)	10 (1.6)	24 (6.5)
Secondary	1818 (23.1)	550 (14.1)	382 (43.7)	265 (18.3)	303 (46.3)	129 (20.3)	189 (51.1)
Higher than secondary	5826 (74)	3296 (84.6)	437 (50)	1158 (79.8)	290 (44.3)	495 (78)	150 (40.5)
Brazilian region							
North	113 (1.4)	38 (1)	15 (1.7)	33 (2.3)	18 (2.8)	6 (0.9)	3 (0.8)
Northeast	842 (10.7)	261 (6.7)	73 (8.4)	213 (14.7)	136 (20.8)	87 (13.7)	72 (19.5)
Center-west	482 (6.1)	226 (5.8)	24 (2.7)	128 (8.8)	42 (6.4)	48 (7.6)	14 (3.8)
Southeast	5498 (69.8)	2798 (71.9)	618 (70.7)	959 (66.1)	408 (62.4)	450 (70.9)	265 (71.6)
South	943 (12)	571 (14.7)	144 (16.5)	118 (8.1)	50 (7.6)	44 (6.9)	16 (4.3)
Living in a state capital							
No	2162 (27.4)	1089 (28)	329 (37.6)	340 (23.4)	181 (27.7)	132 (20.8)	91 (24.6)
Yes	5716 (72.6)	2805 (72)	545 (62.4)	1111 (76.6)	473 (72.3)	503 (79.2)	279 (75.4)
HIV status ^4^							
Negative	5276 (67)	2646 (68)	575 (65.9)	952 (65.6)	426 (65.2)	433 (68.2)	244 (65.9)
Postive	2057 (26.1)	1017 (26.1)	210 (24.1)	413 (28.5)	163 (25)	169 (26.6)	85 (23)
Unknown	543 (6.9)	231 (5.9)	88 (10.1)	86 (5.9)	64 (9.8)	33 (5.2)	41 (11.1)
Recruitment							
Grindr	1699 (21.6)	794 (20.4)	173 (19.8)	346 (23.8)	145 (22.2)	159 (25)	82 (22.2)
Hornet	3488 (44.3)	1854 (47.6)	416 (47.6)	573 (39.5)	268 (41)	227 (35.7)	150 (40.5)
Facebook/Instagram	644 (8.2)	262 (6.7)	91 (10.4)	140 (9.6)	64 (9.8)	47 (7.4)	40 (10.8)
Scruff	2047 (26)	984 (25.3)	194 (22.2)	392 (27)	177 (27.1)	202 (31.8)	98 (26.5)

*IQR* interquartile range

^1^ Missing data = 8

^2^ pansexual, asexual, demisexual, other

^3^ Missing data = 29

^4^ Missing data = 2

Most participants self-identified as White (4758; 60.5%), followed by Pardo (2105; 26.7%) and Black (1005; 12.8%). High-income participants (5980; 75.9%) were followed by low-income (1898; 24.1%) ones. The race-by-income distribution was as follows: high-income White (49.4%), high-income Pardo (18.4%), low-income White (11.1%), low-income Pardo (8.3%), high-income Black (8.1%), and low-income Black (4.7%).

Discrimination was more frequently reported for items 5 (“Called names you do not like”) and 6 (“Excluded/left out by friends”), and less frequently reported for items 4 (“Unfairly evaluated in exams at the workplace”) and 7 (“Excluded/left out by coworkers”) (Table 2). The percentage of participants reporting discrimination across race/income groups differed for all items of the EDS except item 6 (Table 2). For item 1 (“Treated disrespectfully in public places”), 65.7% of high-income and 61.1% of low-income Black participants reported discrimination whereas only 23.2% of high-income and 29.1% of low-income White participants reported discrimination.

**Table 2:** Percentage of participants reporting discrimination for each of the 8 Explicit Discrimination Scale Items, stratified by race/income groups in the cross-sectional, internet-based survey among sexual and gender diverse persons in Brazil, November 2021 to January 2022

	Total n(%)	Race by income intersections						
		White-high	White-low	Pardo-high	Pardo-low	Black-high	Black-low	*p-*value ^1^
		*n* (%)	*n* (%)	*n* (%)	*n* (%)	*n* (%)	*n* (%)	
Item 1: Treated disrespectfully in public places	2593 (32.9)	902 (23.2)	254 (29.1)	548 (37.8)	246 (37.6)	417 (65.7)	226 (61.1)	< 0.001
Item 2: Treated as unintelligent at school/college	1743 (22.1)	619 (15.9)	204 (23.3)	318 (21.9)	195 (29.8)	250 (39.4)	157 (42.4)	< 0.001
Item 3: Treated as unintelligent at the workplace	1699 (21.6)	601 (15.4)	210 (24.0)	327 (22.5)	181 (27.7)	250 (39.4)	130 (35.1)	< 0.001
Item 4: Unfairly evaluated in exams at the workplace	1390 (17.6)	526 (13.5)	184 (21.1)	263 (18.1)	135 (20.6)	178 (28.0)	104 (28.1)	< 0.001
Item 5: Called names you do not like	5109 (64.9)	2471 (63.5)	571 (65.3)	937 (64.6)	404 (61.8)	459 (72.3)	267 (72.2)	< 0.001
Item 6: Excluded/left out by friends at school	3167 (40.2)	1554 (39.9)	356 (40.7)	579 (39.9)	262 (40.1)	266 (41.9)	150 (40.5)	0.960
Item 7: Excluded/left out by coworkers	1467 (18.6)	633 (16.3)	164 (18.8)	289 (19.9)	149 (22.8)	157 (24.7)	75 (20.3)	< 0.001
Item 8: Excluded/left out by people in neighborhood	1530 (19.4)	693 (17.8)	184 (21.1)	289 (19.9)	141 (21.6)	141 (22.2)	82 (22.2)	0.010

^1^ Chi-squared test

Regardless of the item, sexual orientation was frequently reported as a reason for perceived discrimination across the groups (∼62%) (Table 3). Both low- and high-income Black participants frequently reported discrimination based on race (high-income: 76.9%, low-income: 77.3%) and social class (high-income: 48.7%, low-income: 60.0%), whereas high-income White participants reported the lowest frequencies (race: 2.4%, social class: 22.0%). Low-income Black, Pardo and White participants more frequently reported social class and house location as attributions for discrimination when compared to their high-income counterparts. The mean number of attributions for discrimination among low-income Black participants (3.7 SD = 2.1) was higher than that of all other groups (Table 3).

**Table 3:** Reported attributions (absolute numbers and percentages) for discrimination across the 8-items of the Explicit Discrimination Scale stratified by race/income groups in the cross-sectional, internet-based survey among sexual and gender diverse persons in Brazil, November 2021 to January 2022

	Race by income intersections						
	White-high *n* (%)	White-low *n* (%)	Pardo-high *n* (%)	Pardo-low *n* (%)	Black-high *n* (%)	Black-low *n* (%)	*p*-value
Race	93 (2.4)	33 (3.8)	465 (32.0)	219 (33.5)	488 (76.9)	286 (77.3)	< 0.001
Social class	858 (22.0)	297 (34.0)	555 (38.2)	304 (46.5)	309 (48.7)	222 (60.0)	< 0.001
Sexual orientation	2421 (62.2)	559 (64.0)	890 (61.3)	377 (57.6)	390 (61.4)	234 (63.2)	0.200
Gender	531 (13.6)	169 (19.3)	214 (14.7)	125 (19.1)	96 (15.1)	78 (21.1)	< 0.001
Disease	67 (1.7)	38 (4.3)	32 (2.2)	23 (3.5)	6 (0.9)	9 (2.4)	< 0.001
Age	440 (11.3)	126 (14.4)	205 (14.1)	93 (14.2)	105 (16.5)	63 (17.0)	< 0.001
Housing location	202 (5.2)	93 (10.6)	163 (11.2)	100 (15.3)	90 (14.2)	64 (17.3)	< 0.001
Accent	450 (11.6)	154 (17.6)	247 (17.0)	112 (17.1)	79 (12.4)	61 (16.5)	< 0.001
Clothing	55 (1.4)	9 (1.0)	8 (0.6)	6 (0.9)	3 (0.5)	0 (0)	0.012
Overweight	540 (13.9)	129 (14.8)	209 (14.4)	67 (10.2)	83 (13.1)	45 (12.2)	0.11
Physical disability	38 (1.0)	18 (2.1)	20 (1.4)	19 (2.9)	7 (1.1)	11 (3.0)	< 0.001
Appearence	62 (1.6)	21 (2.4)	23 (1.6)	14 (2.1)	2 (0.3)	4 (1.1)	0.034
Political beliefs	413 (10.6)	122 (14.0)	168 (11.6)	64 (9.8)	78 (12.3)	46 (12.4)	0.057
Religion	176 (4.5)	95 (10.9)	100 (6.9)	63 (9.6)	64 (10.1)	42 (11.4)	< 0.001
Other	127 (3.3)	33 (3.8)	28 (1.9)	16 (2.4)	13 (2.0)	6 (1.6)	0.019
Mean number of attributions (SD)	2.4 (1.6)	3.0 (1.9)	3.0 (1.9)	3.2 (1.9)	3.2 (1.8)	3.7 (2.1)	< 0.001

*SD* standard deviation

High-income White participants had the lowest average discrimination score (2.9, SD = 3.2), when compared to all the other groups (*p* < 0.0001 for all comparisons) (Table 4). For Pardo respondents (high-income: 3.5 [SD = 3.6] and low-income: 3.9 [SD = 4.2]) scores varied according to income (low > high). Black participants had the highest average scores regardless of income, with slightly higher SD in the low-income group (high-income: 5.0 [SD = 4.4] and low-income: 5.0 [SD = 4.7]). The mean discrimination score was 1.27 times higher among low-income White and 1.72 times higher among low- and high-income Black respondents, when compared to that of high-income White participants.

**Table 4:** Differences in the mean discrimination score according to the combination race and income in the cross-sectional, internet-based survey among sexual and gender diverse persons in Brazil, November 2021 to January 2022

Race by income intersections	Mean score of discrimination (standard deviation)	Ratio of means ^1^	*p*-value ^1,2^
Overall	3.4 (3.7)	NA	NA
White-high	2.9 (3.2)	NA	NA
White-low	3.7 (4.0)	1.27	< 0.0001
Pardo-high	3.5 (3.6)	1.21	< 0.0001
Pardo-low	3.9 (4.2)	1.34	< 0.0001
Black-high	5.0 (4.4)	1.72	< 0.0001
Black-low	5.0 (4.7)	1.72	< 0.0001

*NA* not applicable

^1^ All comparisons using the White / high category as a reference

^2^ t-student test

Results from the negative binomial regression models revealed significant intersectional patterns of race and income in discrimination experiences that were only slightly altered by adjustment for co-variables (Table 5). In the adjusted model, the joint effect analysis showed that low-income Black participants had discrimination scores 1.78 times higher than high-income White participants (95% CI: 1.63–1.96), while high-income Black participants had scores 1.98 times higher (95% CI: 1.69–2.32). For Pardo participants, low-income individuals had scores 1.37 times higher than high-income White participants (95% CI: 1.27–1.49), and high-income Pardo participants had scores 1.22 times higher (95% CI: 1.09–1.36). Low-income White participants had scores 1.17 times higher than high-income White participants (95% CI: 1.09–1.25). Among other variables, transgender participants had discrimination scores 2.38 times higher (95% CI: 1.76–3.28) and non-binary participants had scores 1.65 times higher (95% CI: 1.38–1.99), both compared to cisgender men, and participants with gay or other sexual orientations had scores 1.20 times higher than bisexual or heterosexual participants (95% CI: 1.11–1.29). Participants with elementary education or lower had scores 0.80 times lower than those with higher than secondary education (95% CI: 0.69–0.94), while age, Brazilian region, living in state capital and HIV status showed no statistically significant associations.

**Table 5:** Results from the negative binomial regression models (exponentiated model coefficients and respective 95% confidence intervals) used to evaluate which factors predict discrimination score in the cross-sectional, internet-based survey among sexual and gender diverse persons in Brazil, November 2021 to January 2022

Total	Model 1	Model 2
Race and Income		
White-high	Ref.	Ref.
White-low	**1.17 (1.10–1.26)**	**1.17 (1.09–1.25)**
Pardo-high	**1.21 (1.08–1.35)**	**1.22 (1.09–1.36)**
Pardo-low	**1.35 (1.26–1.46)**	**1.37 (1.27–1.49)**
Black-high	**1.97 (1.68–2.32)**	**1.98 (1.69–2.32)**
Black-low	**1.81 (1.65–1.98)**	**1.78 (1.63–1.96)**
Co-variables		
Age (years)		
18–24	NA	Ref.
25–35	NA	1.06 (0.97–1.16)
35+	NA	0.92 (0.84–1.01)
Gender		
Cisgender man	NA	Ref.
Transgender person	NA	**2.38 (1.76–3.28)**
Non-binary person	NA	**1.65 (1.38–1.99)**
Sexual Orientation		
Gay or Other ^1^	NA	**1.20 (1.11–1.29)**
Bisexual or Heterosexual	NA	Ref.
Education		
Elementary or lower	NA	**0.80 (0.69–0.94)**
Secondary	NA	0.95 (0.89–1.01)
Higher than secondary	NA	Ref.
Brazilian region		
North, Northeast and Center-west		Ref.
Southeast and South		1.05 (0.98–1.12)
Living in a state capital		
No		Ref.
Yes		1.03 (0.98–1.08)
HIV status		
Negative	NA	Ref.
Postive	NA	1.02 (0.96–1.08)
Unknown	NA	0.91 (0.83–1.01)

^1^ pansexual, asexual, demisexual, other. Bold font indicates statistically signficant results (p<0.05).

**Fig. 2:**
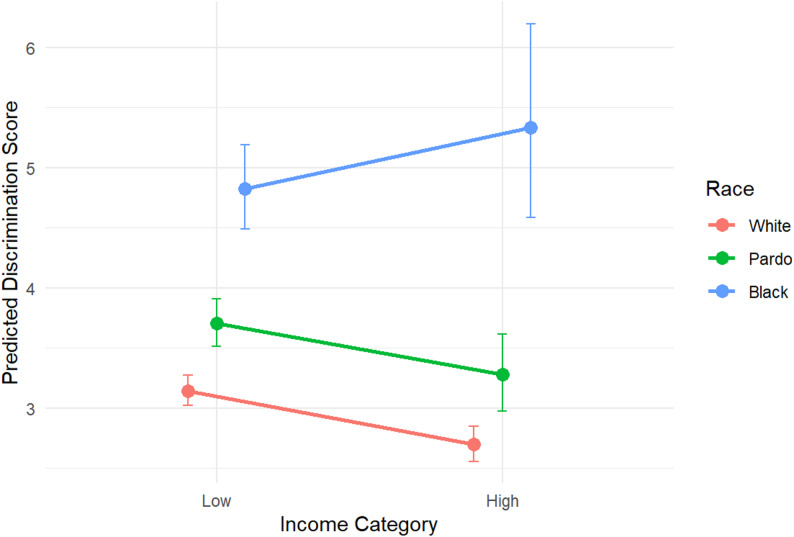
Graphical representation of the joint effect of race and income on discrimination score (measured via the Explicit Discrimination Scale). The final model was adjusted for age, gender, sexual orientation, education, Brazilian region, living in state capital and HIV status

The graphical representation of the race by income interaction shows that among White and Pardo participants, higher income leads to lower discrimination score. In contrast, among Black participants, higher income is associated with a slightly higher discrimination score, though the confidence intervals are wide and overlap (Fig. 2).

## Discussion

This study provides critical insights into the intersectional nature of perceived discrimination among sexual and gender diverse persons in Brazil. The primary findings underscore that race and income are significantly associated with discrimination, with Black participants, irrespective of income, reporting the highest mean discrimination scores. Notably, sexual orientation was consistently identified as a reason for perceived discrimination across all racial and income groups, highlighting the pervasive impact of LGBTQIAPN+phobia. These results emphasize the compounded disadvantages faced by racially and economically marginalized LGBTQIAPN+ populations, calling for targeted public policies that address these intersecting forms of discrimination. By analyzing the socioeconomic and racial disparities within the context of LGBTQIAPN+ populations, this study contributes to a more nuanced understanding of perceived discrimination in Brazil.

One important finding is the consistent reporting of discrimination by Black participants, regardless of income level. This aligns with our previous analysis indicating that race-based discrimination is a primary driver of mistreatment among Black particnts [19]. This persistent discrimination may reflect the historical and ongoing impacts of racism in Brazilian society, where systemic inequalities continue to affect the lived experiences of Black individuals [ipa^6^]. The intersection of race and sexual orientation creates a unique form of oppression, where individuals face discrimination based on both their racial and sexual/gender identities [8]. This is particularly relevant in Brazil, where the legacy of slavery and colonialism has created deep-seated racial hierarchies that continue to shape social, economic, and political opportunities [22]. Studies have shown that Afro-Brazilians experience higher rates of poverty, lower levels of education, and limited access to healthcare compared to their white counterparts [5]. Afro-Brazilians have worse outcomes related to HIV and other sexually transmitted infections care and prevention as well [23–25].

The study also reveals that income disparities play a significant role in shaping experiences of discrimination. Participants of lower-income reported higher levels of discrimination, likely due to reduced access to protective resources such as legal support, social networks, or the ability to relocate from stigmatized areas. This finding is consistent with the broader literature highlighting the ways economic vulnerability amplifies experiences of prejudice and marginalization. A multicenter study with 531 Brazilian dental students found that being Black, female, low-income, or LGBTQIAPN+ increased the likelihood of experiencing discrimination, revealing an intersection of racial, gender, and socioeconomic factors in academic settings [26]. These patterns reinforce the importance of strengthening policies that address structural inequities. Income transfer programs like Bolsa Família have been associated with improved health outcomes, including reduced child mortality and increased access to healthcare [27]. Similarly, affirmative action policies in higher education for students of Black race and low-income not only promote inclusion but have also been linked to long-term health benefits by improving educational attainment and economic stability, key social determinants of health [28].

Results from our regression models also suggest that, among Black participants, the impact of income was reversed from that observed among White and Pardo participants, with the predicted mean score for high-income Black participants being higher than that of low-income ones. Similarly, in a study that compared perceived lifetime discrimination experiences across race, gender, and educational attainment among 13,247 Brazilian and 1,680 U.S. adults, educational attainment was positively associated with reports of discrimination [29]. Though Black participants in both countries, particularly Black men, reported higher levels of discrimination, in Brazil, more educated Black individuals scored higher – a finding that may result from increased awareness or recognition of discriminatory treatment. Acknowledging that our study focuses on income and not education, but at the same time understanding that education and income strongly correlate with one another, our finding might result from the increased awareness of high-income Black participants of discriminatory experiences. That said, in another study of 832 university students from Brazil that assessed experiences of discrimination across intersections of gender, race, and socioeconomic status, high-income participants generally reported lower discrimination scores, regardless of race or gender [30].

While this study provides valuable insights to the literature on perceived discrimination, some limitations should be acknowledged. The cross-sectional design precludes causal inference and prevents assessment of temporal relationships or changes in perceived discrimination over time. Reliance on self-reported data may introduce bias, as participants might underreport or overreport their experiences of discrimination due to social desirability or recall bias. The use of an online survey may also limit the generalizability of the findings, as it excludes individuals with limited internet access, potentially underrepresenting participants with lower-income or those living in more remote areas. Our sample may be subject to selection bias due to the large number of participants who did not complete the 8-items of the EDS, the outcome variable. We have previously noted that a large fraction of participants drop-out from online studies, meaning that they withdraw from the questionnaire before completing it [31, 32]. Prior online surveys among Brazilian MSM have shown lower completion rates among younger participants, those with lower education, and lower income, potentially biasing results toward older, higher-SES individuals [33]. Additionally, potential unmeasured confounders, such as the impact of the COVID-19 pandemic, were not accounted for in this analysis.

## Conclusions

This study corroborates the literature on the pervasive and multifaceted nature of discrimination experienced by sexual and gender diverse persons in Brazil, with disparities according to race and income. The findings highlight the compounded disadvantage faced by Black and low-income individuals, who perceive heightened levels of discrimination across multiple domains. In an era marked by increasing political instability, studies like ours become even more crucial. By providing empirical evidence of the intersectional challenges faced by LGBTQIAPN+ persons in Brazil, this research can inform advocacy efforts and policy interventions aimed at protecting vulnerable communities during times of political upheaval. Ensuring the rights of marginalized populations, particularly Black and low-income LGBTQIAPN+ persons, must be regarded as a central pillar for promoting health equity. Investments in anti-racist education and the protection and expansion of redistributive policies such as Bolsa Família and affirmative action in education are essential strategies to confront the intersecting forms of discrimination that continue to shape health outcomes in the country.

## Data Availability

Study’s final deidentified dataset and dictionary will be made available with publication of the manuscript upon reasonable request. A proposal should be submitted to the corresponding author’s e-mail, who will evaluate and approve the request. No additional documents will be made available.

## Abbreviations

BRL: Brazilian real (currency)
IBGE: Instituto Brasileiro de Geografia e Estatística (Brazilian Institute of Geography and Statistics)
IP: internet protocol
LGBTQIAPN+: Lesbian, gay, bisexual, transgender, queen, intersex, asexual, pansexual, non-binary, plus
MSM: Men who have sex with men
SD: standard deviation
USD: United States dollar (currency)
